# RTF: a rapid and versatile tissue optical clearing method

**DOI:** 10.1038/s41598-018-20306-3

**Published:** 2018-01-31

**Authors:** Tingting Yu, Jingtan Zhu, Yusha Li, Yilin Ma, Jianru Wang, Xinran Cheng, Sen Jin, Qingtao Sun, Xiangning Li, Hui Gong, Qingming Luo, Fuqiang Xu, Shanting Zhao, Dan Zhu

**Affiliations:** 1 Britton Chance Center for Biomedical Photonics, Wuhan National Laboratory for Optoelectronics-Huazhong University of Science and Technology, Wuhan, 430074 China; 2 MoE Key Laboratory for Biomedical Photonics, Collaborative Innovation Center for Biomedical Engineering, School of Engineering Sciences, Huazhong University of Science and Technology, Wuhan, 430074 China; 3 College of Veterinary Medicine, Northwest A&F University, Yangling, 712100 China; 4 Center for Brain Science, Wuhan Institute of Physics and Mathematics, Chinese Academy of Sciences, Wuhan, 430071 China; 5 Center for Excellence in Brain Science and Intelligence Technology, Chinese Academy of Sciences, Shanghai, 200031 China

## Abstract

Tissue optical clearing enables imaging deeper in large volumes with high-resolution. *Clear*^*T2*^ is a relatively rapid clearing method with no use of solvents or detergents, hence poses great advantage on preservation of diverse fluorescent labels. However, this method suffers from insufficient tissue transparency, especially for adult mouse brain blocks. In this work, we develop a rapid and versatile clearing method based on *Clear*^*T2*^, termed RTF (Rapid clearing method based on Triethanolamine and Formamide), aiming for better clearing capability. The results show that RTF can not only efficiently clear embryos, neonatal brains and adult brain blocks, but also preserve fluorescent signal of both endogenous fluorescent proteins and lipophilic dyes, and be compatible with virus labeling and immunostaining. With the good transparency and versatile compatibility, RTF allows visualization and tracing of fluorescent labeling cells and neuronal axons combined with different imaging techniques, showing potentials in facilitating observation of morphological architecture and visualization of neuronal networks.

## Introduction

The development of diverse fluorescent labeling methods and optical imaging techniques have paved the path for three-dimensional reconstruction of tissue structures with high-resolution. However, optical imaging of thick tissues is subjected to high scattering owing to the mismatching of refractive index between different cellular components^1–3^, e.g. the interstitial fluid with low refractive index and collagen fiber with high refractive index.

Tissue optical clearing has emerged to reduce tissue scattering for imaging deep in large volumes^3–9^. Up to now, a variety of optical clearing methods have been developed that significantly promote the development of neuroscience^10–31^. They were generally divided into two categories, including solvent-based and aqueous-based clearing methods. Most of them could preserve endogenous fluorescence of proteins, but were not compatible with lipophilic dyes, which are indispensable for neural tract tracing of post-fixed tissues^14^. Except for the solvent-based 3DISCO^13^ and uDISCO^29^, some aqueous-based methods utilizing high concentration detergents, e.g. CUBIC with Triton X-100^18,19,31^ and CLARITY with sodium dodecyl sulfate (SDS)^16,21^, all realized tissue transparency by lipid removal, hence could not preserve the fluorescent signal of lipophilic dyes. Some other approaches, such as SeeDB^14,17^, FRUIT^25^, *Clear*^*T2*^ ^15^ and Sca*l*eS^23^, were developed to allow preservation of fluorescent signal of lipophilic dyes. Among these techniques, *Clear*^*T2*^ was described as a relatively simple and rapid clearing method to preserve fluorescent signals of lipophilic dyes and immunohistochemistry, as well as fluorescent proteins with no use of solvents or detergents^15^. Nevertheless, the transparency of tissues treated with *Clear*^*T2*^ was not sufficient^32^, especially for adult brain blocks.

In this work, we developed a new clearing method based on *Clear*^*T2*^, termed RTF. It can achieve better transparency in both developing and adult brain tissues while retaining the clearing rapidity. What’s more, RTF shows better fluorescence preservation of endogenous fluorescent proteins, and demonstrates fine compatibility with other diverse labels, including lipophilic dyes, virus labeling and immunostaining. Using RTF, we imaged the hippocampus, embryonic brain and embryo to visualize the neuron distribution and trace the nerve tracts.

## Results

**Figure 1:**
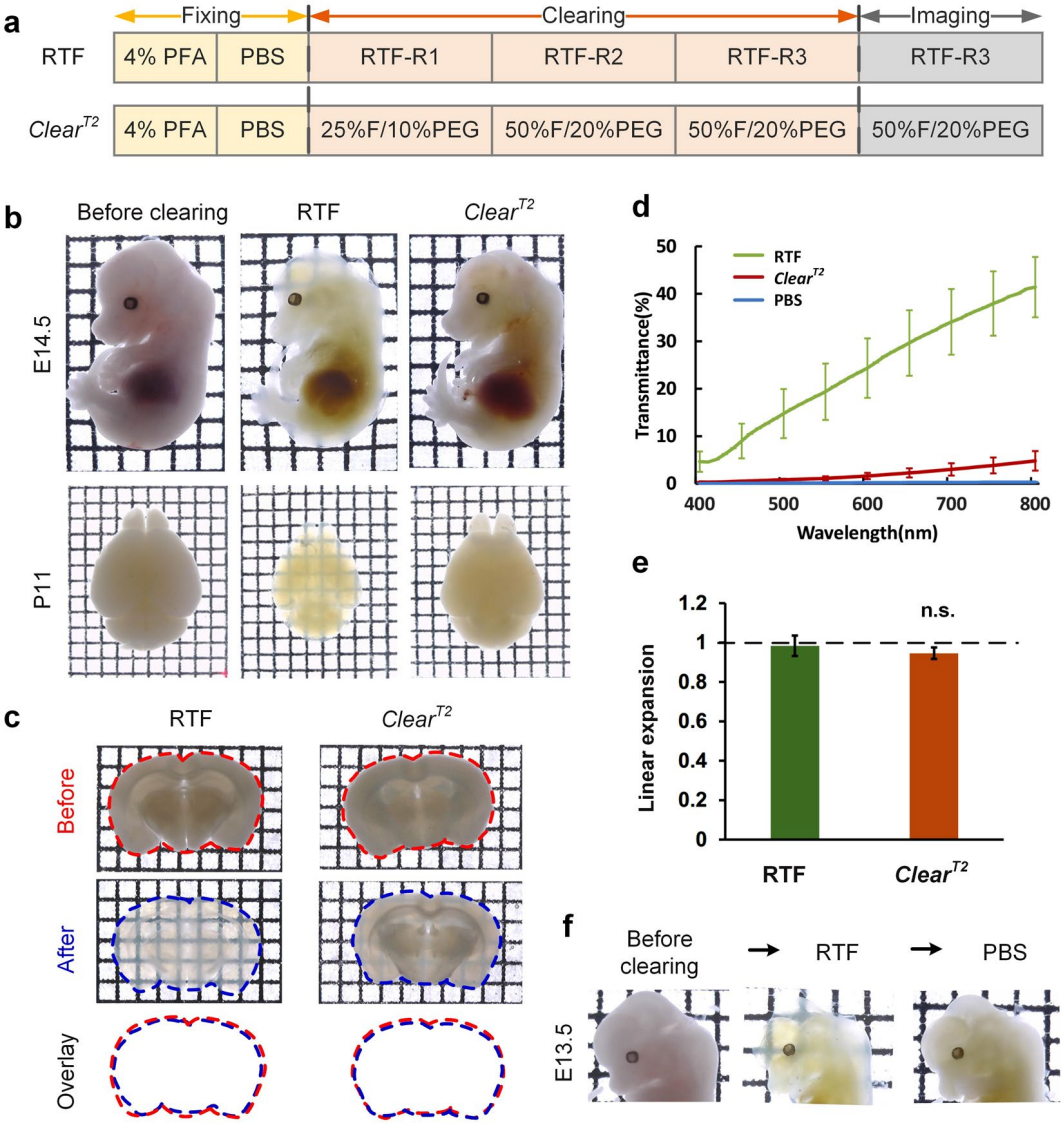
Rapid optical clearing using RTF. (**a**) Clearing steps of RTF and *Clear*^*T2*^. The boxes in the diagram only indicate the step. (**b**) Whole embryos (E14.5) and neonatal (postnatal day 11, P11) whole-brain samples cleared with RTF and *Clear*^*T2*^ overnight (transmission images). Grid size, 1.45 mm × 1.45 mm. (**c**) Adult brain slices (1-mm-thick) cleared with various clearing protocols. The outlines of the brain slices were drawn with dashed lines. Grid size, 1.45 mm × 1.45 mm. (**d**) Transmittance curves of the cleared mouse brain sections (1-mm-thick) (mean ± s.d., n = 6). **(e**) Normalized linear expansion of adult brain slices (1-mm-thick) after optical clearing (mean ± s.d., n = 6). Mann-Whitney U test was used to compare the difference of *Clear*^*T2*^ with RTF. n.s., not significant. (**f**) Clearing of RTF is reversible with PBS. Grid size, 1.45 mm × 1.45 mm.

To develop a rapid optical clearing method for imaging diverse samples, we sought to screen the chemicals from the available clearing techniques in principle of no detergents or solvents. Formamide, as the main effective component in *Clear*^*T2*^, was first chosen for its advantage on clarity and rapidity. Triethanolamine used in CUBIC was also selected due to its high refractive index and suitable alkalinity. The mixture solutions of the two chemicals and water with different ratios constituted the clearing agents of RTF (Supplementary Table 1), the clearing step was shown in Fig. 1a. We first evaluated the clearing performance of RTF on different samples, then investigated the fluorescence preservation, cell morphology maintenance and increase of imaging depth. What’s more, we applied RTF to clear embryonic brain and embryo for visualization of neurons and nerve tracts.

### RTF is a simple and rapid clearing method

Like *Clear*^*T2*^, RTF is a rapid clearing method, it can render the samples transparent within hours to 1 day (Supplementary Table 1). Additionally, it is user-friendly with no need for complicated setups or expensive agents. Here, we demonstrated the effectiveness of RTF, and compared its clearing performance with *Clear*^*T2*^. We processed the fixed intact embryos, neonatal whole brains, and thick adult brain sections with RTF and *Clear*^*T2*^, respectively. The bright-field images showed that RTF rendered the samples more transparent than *Clear*^*T2*^ (Fig. 1b). Even with prolonged treatment with *Clear*^*T2*^ agents, there was no evident increase in samples’ transparency (Supplementary Fig. 1). The transmittance measurement of adult brain sections (1-mm-thick) further quantitatively demonstrated better clearing capability of RTF compared with *Clear*^*T2*^ (Fig. 1d). Though *Clear*^*T2*^ could achieve good transparency on newborn mouse brain slices (Supplementary Fig. 2), it showed limited clearing capability on adult brain tissues while RTF performs well. In addition, the bright-field images demonstrated that RTF showed similar transparency as SeeDB, Sca*l*eSQ(0) and FRUIT, while further quantitative data showed that RTF achieved similar transmittance curve as SeeDB, but lower transmittance value than Sca*l*eSQ(0) and FRUIT (Supplementary Fig. 3b,c).

After the clearing procedure, RTF-treated adult brain section was similar to its original size (0.98 ± 0.05), and the size change showed no significant difference with *Clear*^*T2*^ (0.95 ± 0.03) (Fig. 1c,e) and SeeDB (1.03 ± 0.02) (Supplementary Fig. 3d), while Sca*l*eSQ(0) and FRUIT demonstrated obvious expansion (Sca*l*eSQ(0) = 1.25 ± 0.04; FRUIT = 1.24 ± 0.02) (Supplementary Fig. 3b,d). In addition, the transparency is reversible for RTF (Fig. 1f), and the samples can be transferred from RTF-R3 to PBS to recover to the uncleared state for long-time storage.

### RTF preserves endogenous fluorescence signal and fine structure

**Figure 2:**
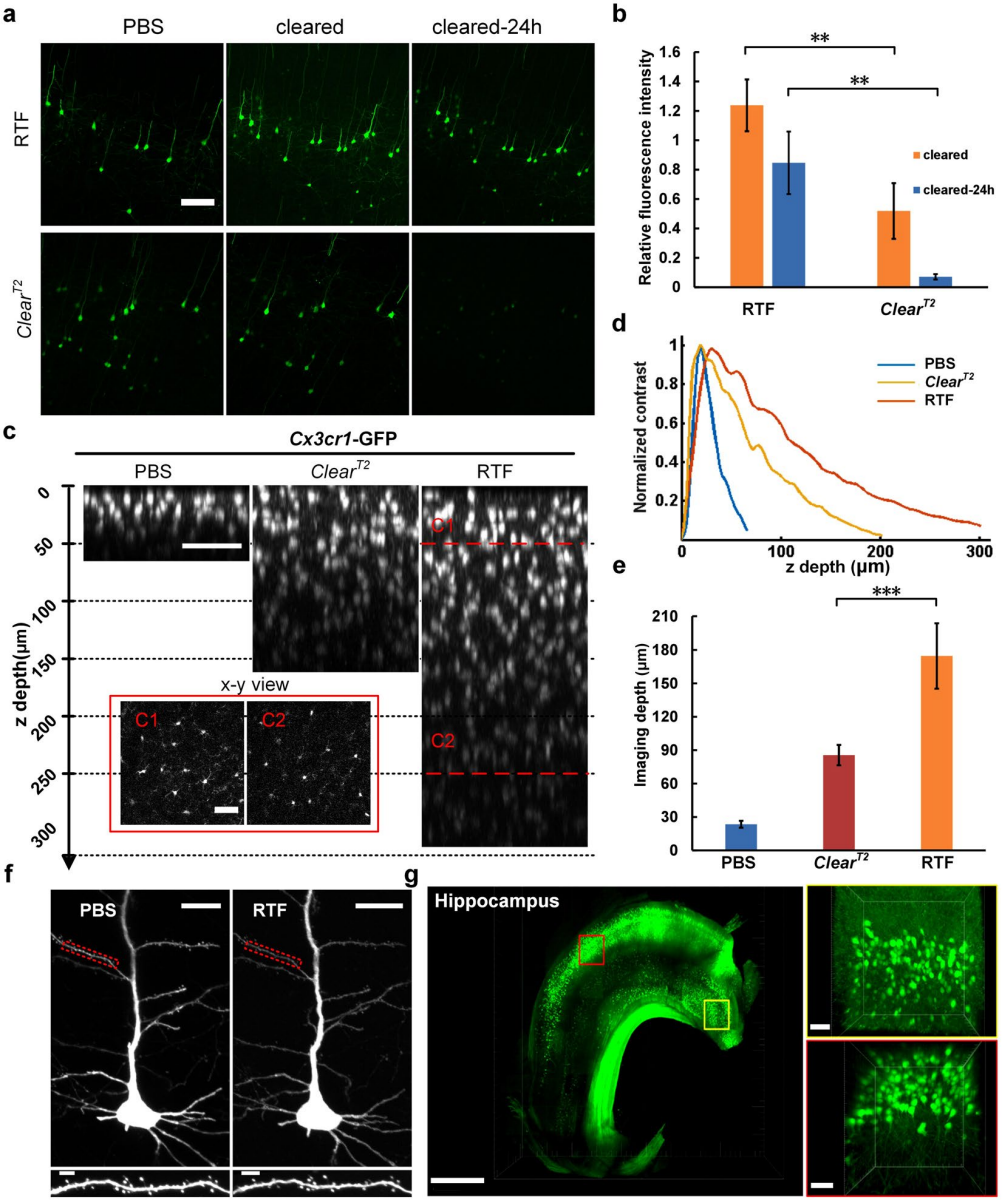
Preservation of GFP fluorescence and morphology maintanence. (**a**) *Thy1*-GFP-M line adult brain slices (1-mm-thick) were prepared and imaged before and after clearing with RTF and *Clear*^*T2*^. Maximum intensity projections of representative images were shown. Scale bar, 100 μm. (**b**) Quantifications of fluorescence intensity for GFP. The relative total fluorescence intensity after RTF clearing showed significantly higher value than *Clear*^*T2*^. Data are presented as mean ± s.d (n = 5). **P < 0.01 (Mann-Whitney U test). (**c**) Orthogonal view (x-z) of image stacks obtained from *Cx3cr1*-GFP mouse brain slices (1-mm-thick). Scale bar, 50 μm. The insets were the optical slices (x-y view) indicated with dashed red line in C. (**d**) Represented curves of image contrast against z depth. (**e**) Bar plot of imaging depth calculated based on contrast decay for PBS, *Clear*^*T2*^ and RTF. Data are presented as mean ± s.d (n = 4). ***P < 0.001 (One-way ANOVA followed by Dunnett’s *post hoc* test). (**f**) Typical pyramidal neurons imaged before and after clearing. Scale bar, 20 μm in top, 5 μm in bottom. (**g**) Reconstruction of neurons in hippocampus of *Thy1*-GFP-M mouse. Scale bar, 1000 μm in left, 50 μm in right.

A critical criterion for a clearing method is whether the clearing solutions are compatible with endogenous fluorescent proteins, such as commonly-used GFP or YFP. Here, we tested RTF on GFP fluorescence and compared with *Clear*^*T2*^, which could effectively maintain the fluorescent signal of genetically encoded proteins. The fluorescent images (*Thy1*-GFP-M) and quantified fluorescence intensity showed that RTF could preserve GFP signal significantly better than *Clear*^*T2*^ (Fig. 2a,b) and allowed storage for longer time. We also tested its performance on YFP signal, and the results also demonstrated fine preservation (Supplementary Fig. 4).

We next compared the imaging depth of brain slices cleared with RTF and *Clear*^*T2*^. *Cx3cr1*-GFP mouse brain slices were used because of its relatively uniform fluorescent cell distribution. Based on the obtained image stacks, we made the orthogonal projection (x-z projection) (Fig. 2c) and calculated the imaging depth based on the contrast decay^26^ (Fig. 2d). The results showed that both methods induced significant increase compared with uncleared state, and RTF achieved about 2-folds imaging depth of *Clear*^*T2*^ (Fig. 2e). Except for the gross size change examined before, we further investigated the influence of RTF on fine structure preservation. To this end, we imaged a typical pyramidal neuron and observed the gross cell morphology and spines on the dendrites before and after clearing. It showed that RTF could maintain the cell morphology and fine structures well (Fig. 2f, Supplementary Fig. 5). Afterwards, the dissected hippocampus of *Thy1*-GFP-M mouse was cleared with RTF and imaged with Ultramicroscope. The reconstructed three-dimensional structures provided clear visualization of the neurons (Fig. 2g).

### RTF is compatible with virus labeling, immunostaining and DiI-labeling

Except for the transgenic fluorescent proteins, other cell labeling strategies, such as virus labeling, immunostaining and dyes (stains) labeling, are also valuable and commonly used for labeling molecules of interest in many studies. To examine whether these labeling strategies are compatible with RTF clearing, we labeled the brain tissue with rabies virus, antibodies against GFP and DiI, respectively.

**Figure 3:**
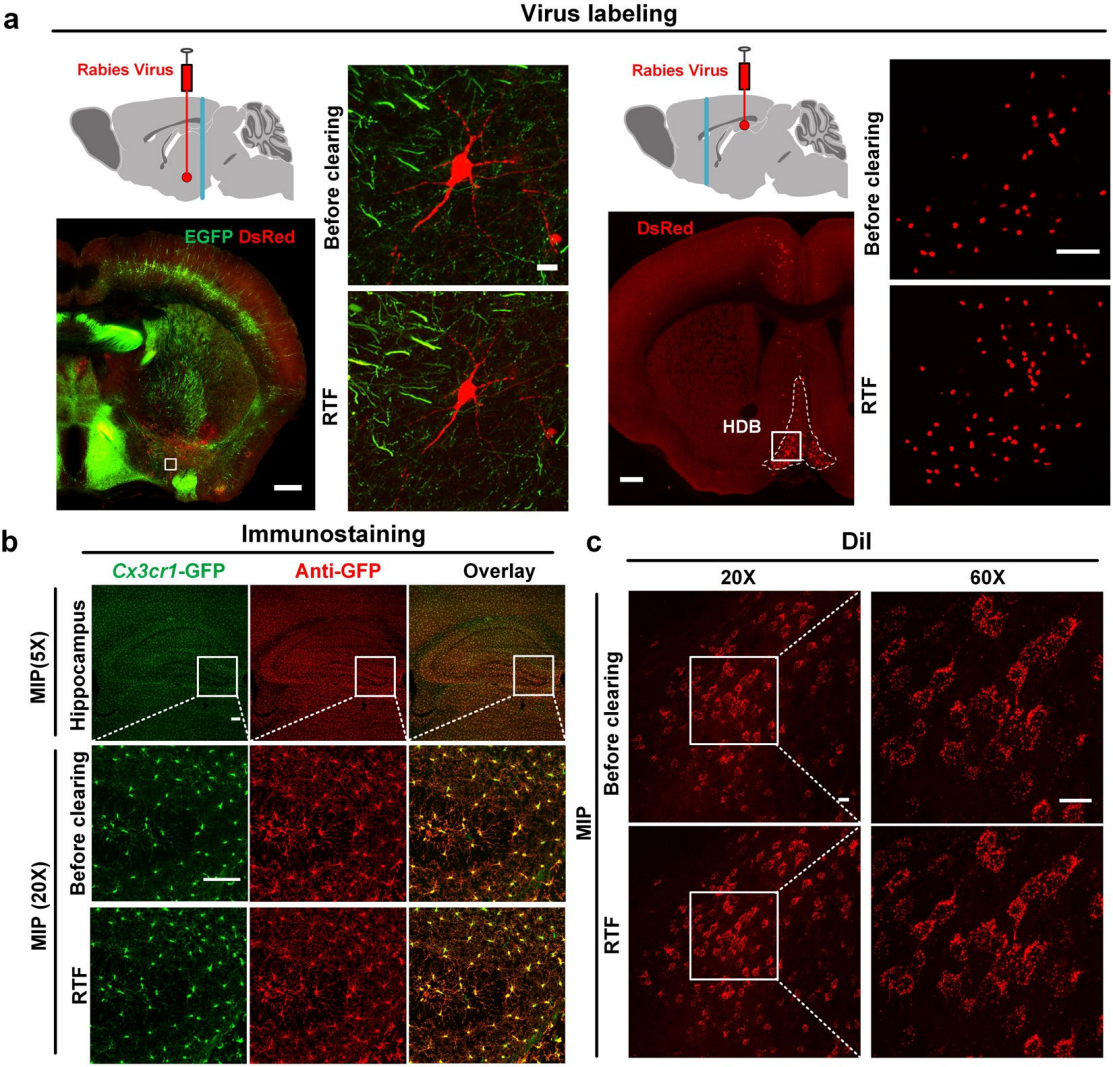
Compatibility of RTF with virus labeling, immunostaining and DiI-labeling. (**a**) The injection site of rabies virus (red point) and position of brain section (cyan line) are shown in the schematic diagram. This diagram was drawn by referring to The Mouse Brain in Stereotaxic Coordinates, 2nd edition, Franklin, K.B.J. and Paxinos, G. After transducing with RV-ΔG-dsRed, the brain sections at indicated positions were dissected and imaged before and after clearing with RTF. The maximum intensity projections of enlarged region are indicated with white box in the image of half brain section. DsRed in red and EGFP in green. The images showed that endogenous GFP and virally delivered dsRed expression both presented fine fluorescent signal. Scale bar, 500 μm for first and third panel; 50 μm for second panel; 100 μm for fourth panel. (**b**) *Cx3cr1*-GFP hippocampus sections immunolabeled with microglial cells marker anti-GFP, cleared with RTF; The GFP and anti-GFP fluorescence (with a secondary antibody conjugated to Alexa Fluor 594) were visualized. Scale bar, 100 μm. (**c**) Merged stack of DiI-labeled cells in MD region imaged from 400 μm brain section, before and after clearing. Scale bar, 20 μm.

The labeled neurons with RV-ΔG-dsRed injection at BLA (basolateral amygdaloid nucleus) showed fine fluorescent signal after clearing with RTF, as well as transgenic GFP. The fluorescence expression of virally delivered proteins in HDB (nucleus of the horizontal limb of the diagonal band) was also detectable with strong intensity by injection of RV-ΔG-dsRed in CA1 (cornu ammonis areas) (Fig. 3a). After RTF clearing, the signals from immunostained GFP were still visible and overlaid well with the endogenous GFP signal (*Cx3cr1*-GFP), indicating fine compatibility of RTF with immunolabeling (Fig. 3b). Additionally, we used DiI, a powerful neuronal tracer, to retrogradely label the mediodorsal thalamic nucleus (MD) in adult mouse brain. The labeled slices were cleared with RTF, and the results demonstrated the important advantage of RTF in preserving lipophilic fluorescent dyes (Fig. 3c).

### RTF enables visualization of axons in intact embryos and neurons in embryonic brain

**Figure 4:**
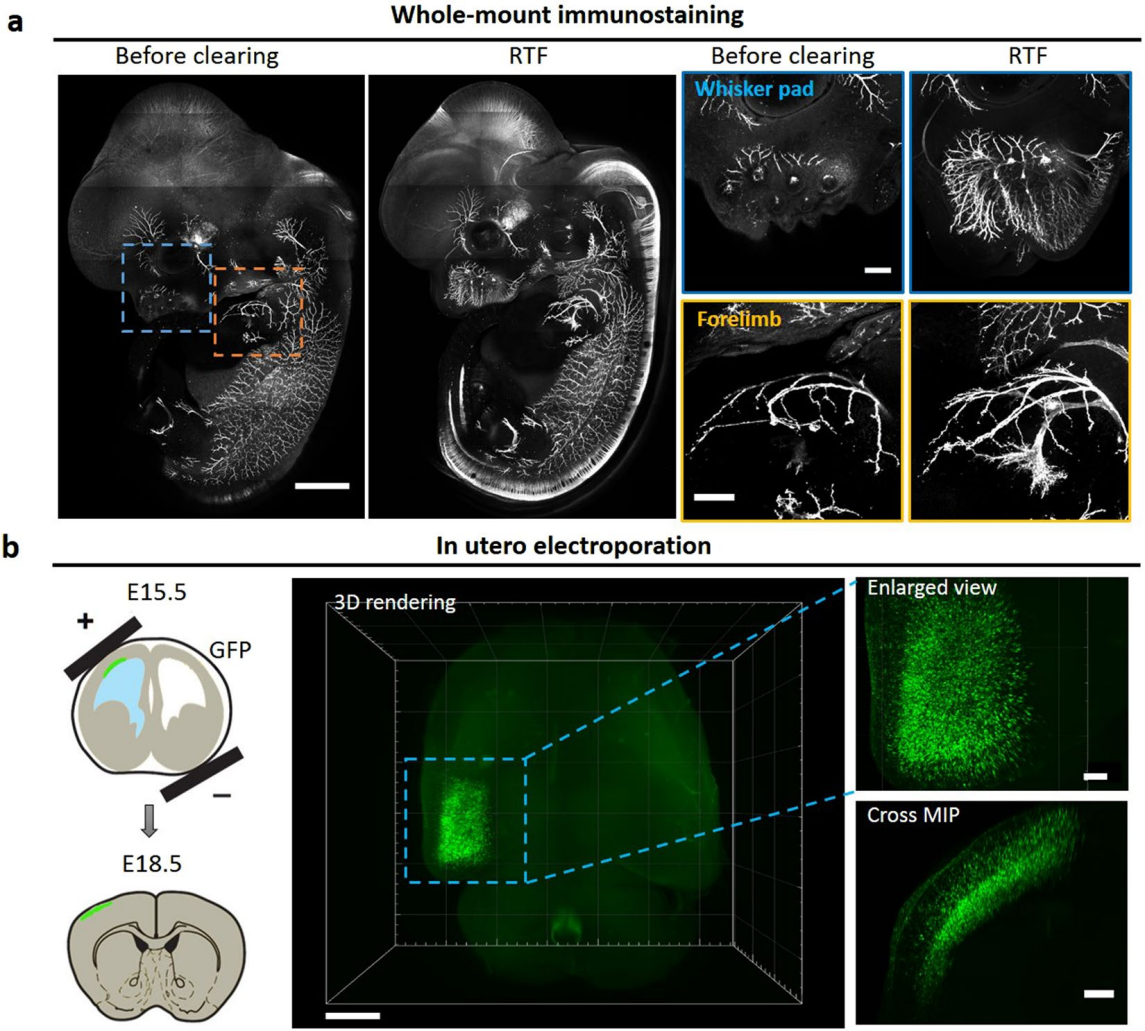
Visualization of axons in whole-mount embryo and neurons in embryonic brain by RTF clearing. (**a**) E12.5 whole mouse embryo was immunostained with neurofilament antibody and cleared with RTF. The axons were much more visible after clearing; magnification of forelimb whisker pad (blue box in dashed line) and (yellow box in dashed line) demonstrate much more details by RTF clearing. Scale bar, 1000 μm in left two images, 200 μm in right four images. (**b**) After IUE at E15.5, the brain was harvested at E18.5. After clearing with RTF and imaging with Ultramicroscope, the transfected neurons in whole brain were reconstructed in three-dimension; the enlarged view of 3D rendering and maximum projection in cross section demonstrate the localization of the neurons in cortex. Scale bar, 1000 μm in left (3D rendering), 200 μm in right (Enlarged view and Cross MIP). The schematic representation of IUE was drawn by referring to ref.^37^.

We used RTF on intact mouse embryo and embryonic brain for visualization of nerves and neurons deep inside tissues. The whole-mount embryo was immunostained with antibody to neurofilament (anti-NF), then cleared with RTF and imaged with confocal microscopy. The cleared embryo showed a more complete view of the axon tracts and arbors in the nervous system. We could visualize and obtain more information deep in tissues from the enlarged region of whisker pad and forelimb to demonstrate the projection and innervation details (Fig. 4a). Furthermore, we used RTF to clear the embryonic brain, which are commonly used for neural development studies. After employing in utero electroporation (IUE), the brains were harvested, cleared using RTF, and imaged with Ultramicroscope. The reconstruction and resampling images demonstrated the distribution of transfected neurons in three-dimension (Fig. 4b).

## Discussion

In recent years, a variety of optical clearing methods have been proposed to reduce light scattering and facilitate deep imaging in large volumes. As a relatively rapid clearing protocol with no use of solvents and detergents, *Clear*^*T2*^ has excellent compatibility with diverse fluorescent labels, including the lipophilic dyes, but shows insufficient tissue transparency. To address this problem, we developed a clearing method, RTF, which consisted of triethanolamine and formamide. Following a series of evaluation of tissue transparency, fluorescence intensity, imaging depth and cell morphology, RTF is demonstrated to be an efficient and versatile optical clearing method for multiple tissues and labels.

Though there are a number of optical clearing methods having strong clearing capability, many of them are not compatible with lipophilic dyes due to the use of solvents or detergents, such as 3DISCO^13^, CLARITY^16,21^ and CUBIC^18,19,31^. While RTF shows good capability in preserving both the fluorescent signal of genetically encoded proteins (Fig. 2a,b) and the lipophilic dyes (Fig. 3c) like some other detergent- and solvent-free clearing methods, such as *Clear*^*T2*^ ^15^, SeeDB^14,17^, Sca*l*eSQ(0)^23^ and FRUIT^25^. Compared with these methods, RTF provides certain advantages. As described above, RTF demonstrated better tissue transparency and fluorescence preservation than *Clear*^*T2*^ by taking similar clearing time (Supplementary Table 1). The better clearing capability might be due to the higher refractive index of RTF clearing agents (RI = 1.46 for RTF versus 1.41 for *Clear*^*T2*^) (Supplementary Table 2), while the better GFP fluorescence preservation might be owing to the alkalinity of triethanolamine in RTF solutions. This basic property is of benefit to many fluorescent proteins^33^ which are highly pH sensitive (Supplementary Table 2). In addition, the low-viscosity and high-diffusion velocity of RTF agents allows a faster clearing procedure compared with SeeDB^14^ or FRUIT^25^. Sca*l*eSQ(0)^23^, another rapid clearing protocol, can cause obvious expansion like FRUIT^25^, while RTF induces minimal size change as SeeDB^14^ with good morphology maintanence (Supplementary Fig. 3b,d). Furthermore, this clearing procedure has no need for complicated setups and requires only economic reagents, and hence is a user-friendly method. Nevertheless, the tissue transparency of RTF is not as good as many other protocols, and RTF is only suitable for small adult brain blocks, further optimized or new protocols should be developed in the future work to achieve rapid and efficient transparency of larger brain tissues, which is the direction we are working on.

Although we quantified the linear deformation of RTF-cleared brain slices (Fig. 1e), the value is not applicable to all tissue types due to the differences of sample composition and structure^32^. The size change depends on the sample features (e.g. size and age) and incubation time in each solution. If the RTF-cleared samples shrink more than you required, longer incubation time in RTF-R1 (as described in Methods) will diminish this size reduction. Generally, the embryonic tissue with loose structures will experience more shrinkage or expansion during many clearing protocols.

It should be noted that, the original paper of *Clear*^*T2*^ showed the clearing results of 800-μm-thick brain slices of P0 mice, while the results in this paper gave the results of 1-mm-thick brain slices of adult mice. *Clear*^*T2*^ can achieve good transparency on P0 mouse brain slices (Supplementary Fig. 2), but not on adult brain tissues (Fig. 1c). In addition, even if we conducted the experiments strictly according to its protocol and repeated many times, the visual transparency of *Clear*^*T2*^ treated samples still show differences with the original *Clear*^*T2*^ paper. This might be due to the different imaging system for acquiring bright-field images. A Zeiss dissecting microscope was used in the *Clear*^*T2*^ original paper, while a normal camera above combining the illumination under the samples with white light was used in this paper. The brightness and contrast could also influence the visual effect of images. Additionally, the blood residual in samples and the post-fixation degree might also be the reasons. In another published paper^32^, the results of mouse embryos cleared with *Clear*^*T2*^ also did not show the transparency as good as the original *Clear*^*T2*^ paper. So conducting the imaging for *Clear*^*T2*^ and RTF simultaneously in same condition would be necessary and sufficient for the comparison of two methods.

Using RTF, we can achieve visualization of nerves and neurons in embryos and embryonic brain tissues. Hence, RTF is potential to be applied to observe the neural development during different stages. For instance, combining with PI or DAPI counterstaining of cell nucleus for cytoarchitecture, RTF is expected to help determine the neuron localization in certain cortex layer, which could potentially provide full-scale information and be useful for study of neuronal migration.

In a word, this work introduces RTF, a rapid and versatile clearing method with increased tissue transparency and higher fluorescence intensity compared with *Clear*^*T2*^, providing an alternative for brain tissue clearing. But there is much more to do for better clearing capability in the future.

## Conclusion

We developed a new clearing method based on *Clear*^*T2*^, termed RTF. By evaluating from tissue transparency, fluorescence intensity, imaging depth and cell morphology, we demonstrated that RTF could achieve efficient transparency for diverse tissue samples, including embryos, neonatal brains and adult brain blocks, while retaining the clearing rapidity. With better fluorescence preservation of endogenous proteins and fine compatibility with various fluorescent labels, such as lipophilic dye tracers, virus labeling and immunostaining, RTF allows visualization and tracing of fluorescent labeling cells and neuronal axons combined with different imaging techniques. Thus, as a rapid and versatile optical clearing method for multiple tissues and labels, RTF provides an alternative protocol for researchers and shows potential in facilitating observation of morphological architecture and visualization of neuronal networks.

## Methods

### RTF solutions

RTF solutions were prepared by mixing triethanolamine (Sinopharm Chemical Reagent Co., Ltd, China), formamide (Sinopharm Chemical Reagent Co., Ltd, China), and distilled water by volume. A 30%TEA/40%F/30%W mixture refers to 30% triethanolamine (TEA), 40% formamide (F), 30% water (W) solution, termed as RTF-R1. A 60%TEA/25%F/15%W mixture refers to 60% triethanolamine (TEA), 25% formamide (F), 15% water (W) solution, termed as RTF-R2. A 70%TEA/15%F/15%W refers to 70% triethanolamine (TEA), 15% formamide (F), 15% water (W) solution, termed as RTF-R3.

### Preparation of samples

Wild-type (C57BL/6 N) mice (Experimental Animal Center of Wuhan University, China), *Thy1*-GFP-M line mice, *Thy1*-YFP-H line mice and *Cx3cr1*-GFP line mice (Jackson Laboratory, USA) were used in this study. The adult mice used here were 8- to 11-weeks-old. Mice were anesthetized with a mixture of 2% α-chloralose and 10% urethane (8 mL/kg). Wild-type mouse embryos were removed from the anesthetized mothers; the heads of postnatal day 0 (P0) mice were removed and flushed with PBS followed by PFA fixation. P11 mice were perfused intracardially with 0.01 M phosphate buffered saline (PBS, Sigma) followed by 4% paraformaldehyde (PFA, Sigma-Aldrich) in PBS for fixation. Excised mouse embryos or brains were post-fixed overnight in 4% PFA at 4 °C. Coronal brain blocks were sliced using a commercial vibratome (Leica VT 1000 s, Germany). The hippocampus was dissected from fixed *Thy1*-GFP-M line mouse brain. All animal care and all experimental protocols were in accordance with the Experimental Animal Management Ordinance of Hubei Province, P. R. China and the guidelines from the Huazhong University of Science and Technology, and have been approved by the Institutional Animal Ethics Committee of Huazhong University of Science and Technology.

### Optical clearing using RTF

For RTF, the samples were incubated in RTF-R1, RTF-R2 and RTF-R3 sequentially. Incubation time in each solution depends on tissue type and thickness. For whole embryos or heads (E11-E15), it requires 2–3 hr, 2–3 hr, 5–14 hr for RTF-R1, RTF-R2, RTF-R3, respectively. For intact brains (E16-P12), the incubation time in the final solution increases to overnight or longer. For adult brain sections with the thickness of 800–1500 μm, 30–50 min, 1–1.5 hr and 1–1.5 hr in RTF-R1, RTF-R2, RTF-R3 will be enough for clearing (see Supplementary Table 1 for details).

### Optical clearing using *Clear*^*T2*^

*Clear*^*T2*^ has been described previously^15^. For *Clear*^*T2*^, the samples were transferred to 25% formamide/10% polyethylene glycol (PEG) solution, 50% formamide/20% PEG solution, then 50% formamide/20% PEG solution. The incubation time for each method was conducted as the original paper. All other clearing methods, including SeeDB, Sca*l*eSQ(0) and FRUIT (Supplementary Fig. 3a), the clearing protocols were carried out following the original papers^14,23,25^.

### Immunostaining

The adult *Cx3cr1*-GFP mouse brain slices (50-μm-thick) were incubated with blocking solution (0.01 M PBS/10% normal donkey serum/0.1% Triton X-100) for 1 hr at room temperature. The slices were transferred to primary antibody (anti-GFP, Aves, GFP-1020, 1:250) dilutions for overnight at 4 °C followed by washing with PBST (0.1% Triton X-100 in PBS) for three times (5 min/each time), then to secondary antibody dilutions (donkey anti-chicken Alexa Fluor 594, Jackson Immunoresearch, 117978, 1:500) for 2 hr at room temperature, then washed in PBS for several times.

The whole embryos (E12.5) was immunostained for neurofilament based on iDISCO protocol with methanol pretreatment^34^. The pretreated sample was incubated in PBS/0.2% Triton X-100/20% DMSO/0.3 M glycine overnight at 37 °C, blocked in PBS/0.2% Triton X-100/10%DMSO/6% Goat Serum for 1d at 37 °C. After transferring to primary antibody dilutions (anti NF-200, DHSB, 2H3, 1:100) for 3d followed by washing, and then incubated in secondary antibody dilutions (goat anti-mouse Alexa Fluor 594, Jackson Immunoresearch, 115–585–146, 1:300) for 2d at room temperature and finally washed for 1d prior to clearing and imaging.

### Virus labeling

Rabies virus expressed dsRed (RV-ΔG-dsRed) was used here. For the virus injection, 2–3 months old *Thy1*-GFP-M line mice and C57BL/6 N mice were anesthetized and placed in a stereotaxic frame (RWD Lifescience, China). To expose the cortex targeted for tracing neurons, a cranial window on the skull was created. RV-ΔG-dsRed was injected into the cortex using a custom-established injector fixed with a pulled glass pipette. Firstly, the injection sites were targeted with the following coordinates: 3.25 mm lateral; 4.85 mm deep; 1.4 mm posterior to bregma for *Thy1*-GFP-M line mice, and 1 mm lateral; 1.5 mm deep; 1.7 mm posterior to bregma for wild-type C57BL/6 N mice. Next, each virus (300 nl) was injected at a rate of approximately 30 nl/min for the respective injection site. The glass pipette was kept *in situ* for 10 min before moving outside. After finishing the injections, the cranial windows were covered, and the incisions were closed. The animals were placed in a warm cage for waking up and then transferred into a regular keeping room. The animal survived for 1 week post virus injection before performing clearing.

### In utero electroporation

The expression plasmid (pCAG-MCS-EGFP) was constructed by Shanting Zhao’s lab. Plasmids used for IUE were purified with Qiagen Plasmid Plus Midi Kit (Hilden, Germany). IUE was performed as described previously^35–37^. Briefly, pregnant mice were anesthetized with sodium pentobarbital and the uterine horns were exposed. Plasmid mixed with 0.1% Fast Green dye (Sigma) was injected into the embryos’ lateral ventricle with a glass micropipette at E15.5 with five electrical pulses (30 V; 50-ms duration; 950-ms intervals). Then the embryos were placed back into the abdominal cavity for normal development. Then the brains were harvested at E18.5.

### Imaging

For evaluating the clearing capability, the bright-field images of the samples, including embryos, whole-brains of neonatal mice, and brain sections were taken with a camera before and after clearing. The imaging of brain sections and whole embryos with immunolabeling were performed using inverted confocal fluorescence microscopy (LSM710, Zeiss, Germany). A 5× objective lens (FLUAR, NA = 0.25, W.D. = 12.5 mm), a 10× objective lens (FLUAR, N.A. = 0.5, W.D. = 2 mm), a 20× objective lens (PLAN-APOCHROMAT, N.A. = 0.8, W.D. = 550 μm) or a 40× objective lens (PLAN- APOCHROMAT, N.A. = 1.4, W.D. = 130 μm) were used for imaging. The samples were placed on a coverslip and covered with another coverslip to keep tissue submerged in solutions. Imaging of hippocampus and embryonic whole brains were performed using light-sheet microscopy (Ultramicroscope, LaVisionBioTec, Germany) equipped with a 2× objective lens (MVPLAPO) and macro zoom body (magnification steps from 0.63× to 6.3×).

### Measurement of light transmittance

Transmittance curves of cleared brain sections were acquired using a commercially available spectrophotometer (Lambda 950, PerkinElmer, USA). The adult brains sections (1-mm-thick) were placed in customed slides with a 2 mm × 4 mm slit to obtain the collimated transmittance spectra (400–800 nm) due to the small size of brain slices.

### Data processing

The images were processed with ImageJ software and reconstructed with Imaris software. For measurement of size change, based on the bright-field images, the area of the samples were outlined with ImageJ software. The linear expansion value was quantified by normalizing the area of the cleared brain slices with the area of uncleared ones in PBS, and calculating the square root. For fluorescence quantification, the brain slices of *Thy1*-GFP-M line mice were used. The brain sections (1-mm-thick) were imaged before and after clearing with RTF, *Clear*^*T2*^. The mean fluorescence intensity was measured using the freehand-selection tool and histogram tool in ImageJ, and normalized to the intensity value in uncleared state. Data were analyzed and graphs constructed using Matlab, Microsoft Excel or Visio.

### Statistical analysis

Data are presented as mean ± s.d. in all figures. Statistical analyses were performed using GraphPad Prism 5. Mann-Whitney U test is a nonparametric test used to compare two independent groups and does not require the assumption of normal distributions and homogeneity of variances. One-way ANOVA is usually being used to compare at least three groups and requires normal distributions of data. Here, Mann-Whitney U test was used in Figs 1e and 2b due to the unequal variances of data. One-way ANOVA was used in Fig. 2e for comparison of imaging depth in three groups.

### Data availability

The datasets generated and analysed during the current study are available from the corresponding author on reasonable request.

## Electronic supplementary material

Supplementary Information
