# Supplementary material for: RTF: a rapid and versatile tissue optical clearing method
Source: Sci Rep. 2018 Jan 31;8:1964. doi: 10.1038/s41598-018-20306-3 (PMC5792593; doi:10.1038/s41598-018-20306-3)
Supplement: Supplementary file 1 — Supplementary Information [file 41598_2018_20306_MOESM1_ESM.pdf]

Supplementary Information

RTF: a rapid and versatile tissue optical clearing method

Tingting Yu [1,2], Jingtan Zhu [1, 2], Yusha Li [1,2], Yilin Ma [1,2], Jianru Wang [1,2], Xinran Cheng [3], Sen Jin [4], Qingtao Sun [1,2], Xiangning Li [1,2], Hui Gong [1,2], Qingming Luo [1,2], Fuqiang Xu [1,4,5], Shanting Zhao [3], Dan Zhu [1,2,*]

[1]Britton Chance Center for Biomedical Photonics, Wuhan National Laboratory for Optoelectronics-Huazhong University of Science and Technology, Wuhan, 430074, China

[2]MoE Key Laboratory for Biomedical Photonics, Collaborative Innovation Center for Biomedical Engineering, School of Engineering Sciences, Huazhong University of Science and Technology, Wuhan, 430074, China

[3]College of Veterinary Medicine, Northwest A&F University, Yangling, 712100, China

[4]Center for Brain Science, Wuhan Institute of Physics and Mathematics, Chinese Academy of Sciences, Wuhan, 430071, China

[5]Center for Excellence in Brain Science and Intelligence Technology, Chinese Academy of Sciences, Shanghai, 200031, China

*Corresponding author: Dan Zhu, Ph.D, Professor

Email: dawnzh@mail.hust.edu.cn

Tel: +86 27 87792033, Fax: +86 27 87792034

Supplementary Figure 1

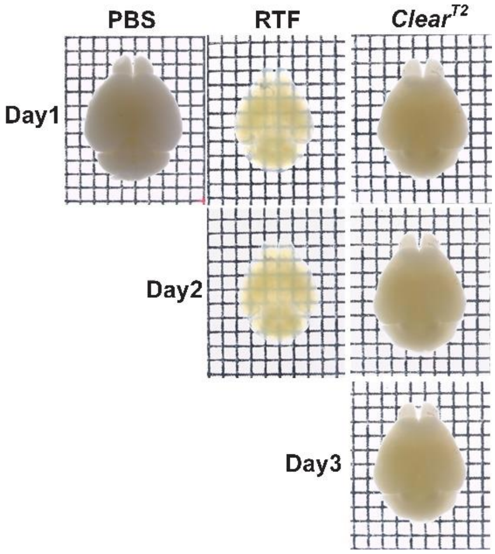

Supplementary Figure 1. Transparency of neonatal whole-brain (P11) treated with each optical clearing method along with time. Grid size, 1.45 mm × 1.45 mm. The transparency achieved with RTF is better than *Clear*^{T2}, even with longer incubation.

Supplementary Figure 2

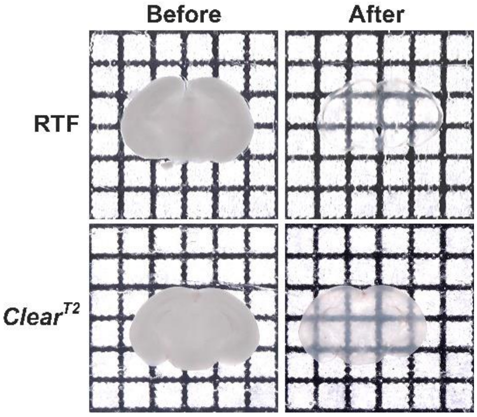

Supplementary Figure 2. Transparency of neonatal 800-µm-thick brain slices (P0) treated with RTF and *Clear*^{T2}. Grid size, 1.45 mm × 1.45 mm. The transparency achieved with *Clear*^{T2} is close to the original paper, and RTF shows better transparency on newborn mouse brain slices than *Clear*^{T2}.

Supplementary Figure 3

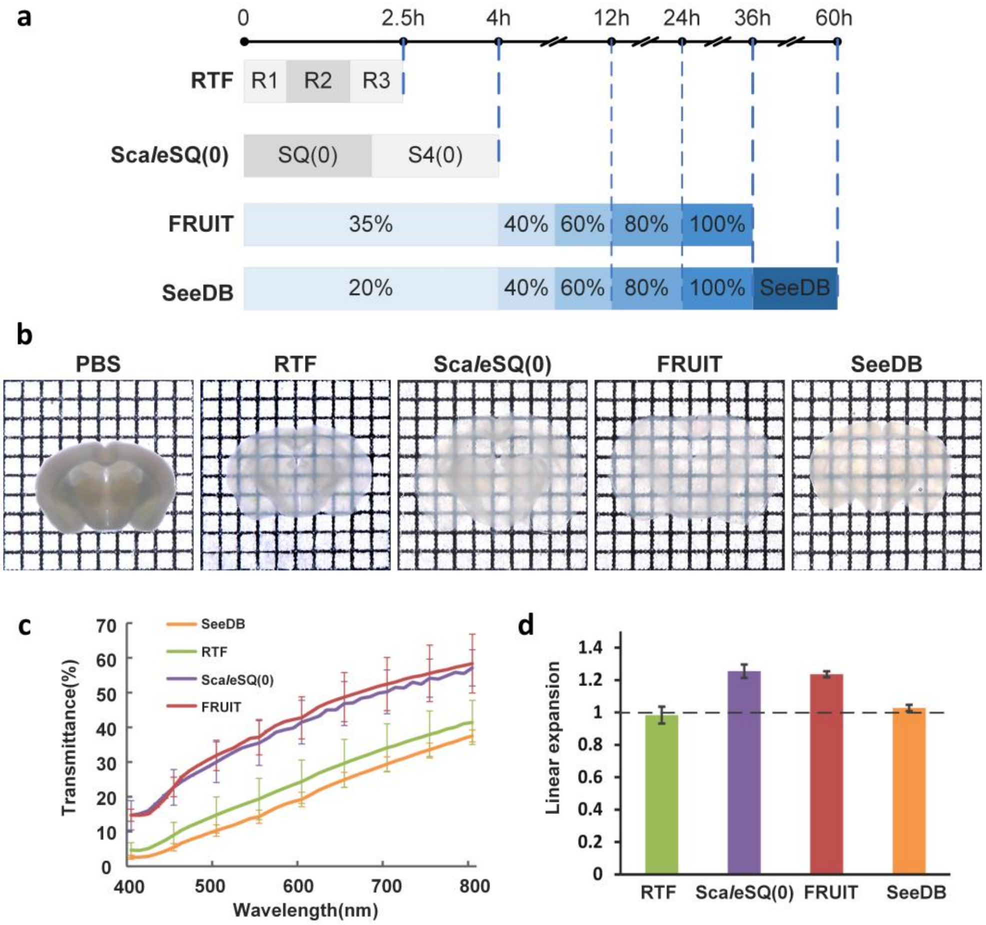

Supplementary Figure 3. Comparison with other detergent- and solvent-free clearing methods. (**a**) Clearing procedures of different clearing methods. RTF requires less incubation time. (**b**) Transparency of 1-mm-thick adult brain slices treated with each optical clearing method. Grid size, 1.45 mm × 1.45 mm. (**c**) Transmittance curves of cleared brain slices (1-mm-thick). For 1-mm-thick brain slices, RTF can achieve similar transparency with SeeDB, but lower transparency than Sca*l*eSQ(0) and FRUIT. (**d**) Normalized linear expansion of adult brain slices (1-mm-thick) after optical clearing. Sca*l*eSQ(0) and FRUIT induce obviously expansion of brain slices, while RTF and SeeDB cause minimal size changes.

Supplementary Figure 4

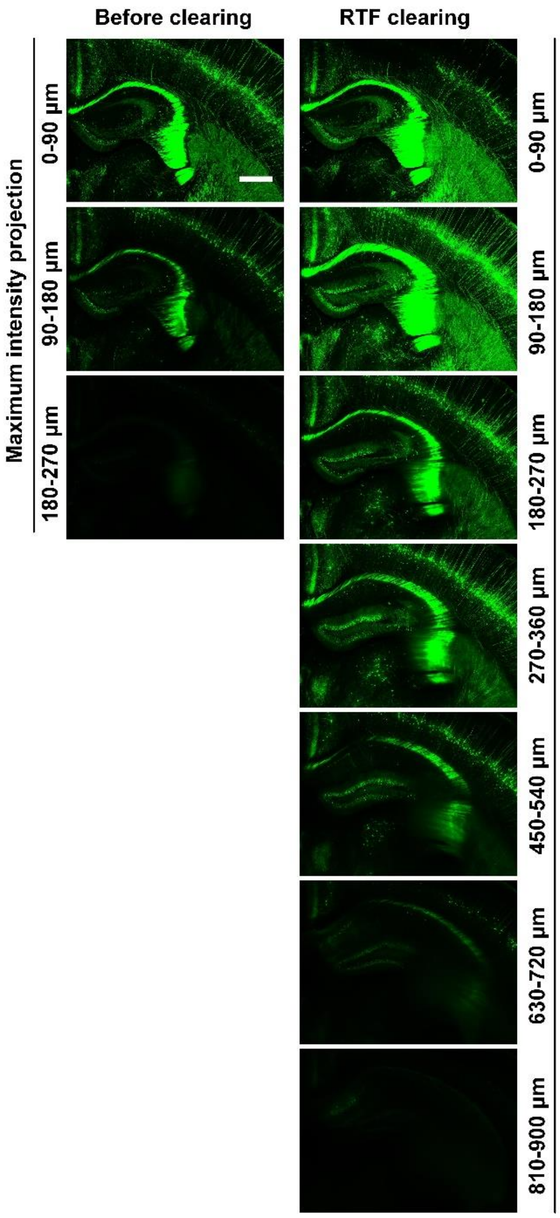

Supplementary Figure 4. YFP is preserved well after clearing with RTF. The mouse brain blocks (*Thy1*-YFP-H) containing part of hippocampus were imaged with 10× objective in confocal microscopy before and after clearing with RTF. The images are maximum intensity projection of 15 images (z step, 6 µm). With fine preservation of YFP fluorescent signal, RTF allows imaging deeper in brain tissues. Scale bar, 500 µm.

Supplementary Figure 5

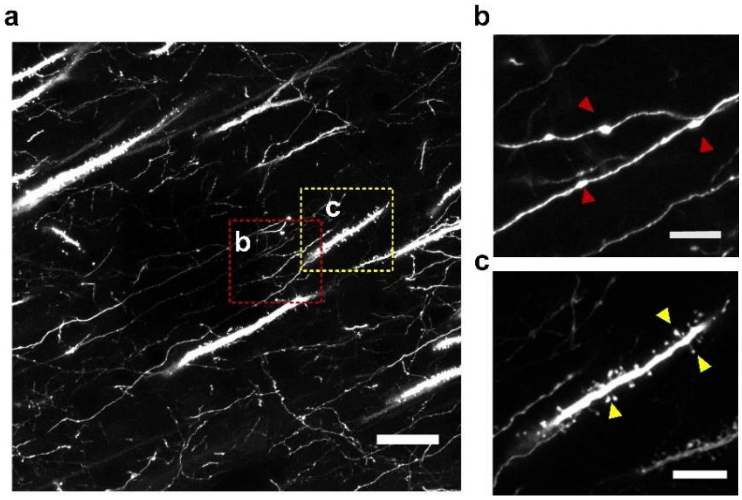

Supplementary Figure 5. Fine structures are preserved after clearing with RTF. (**a**) Maximum intensity projection of image stacks (thickness, 5 µm) in the region of mouse cortex (*Thy1*-YFP-H). Scale bar, 30 µm. (**b**) Image of axons. The buttons (indicated with red triangles) are clearly visible. Scale bar, 10 µm. (**c**) Image of dendrites. The spine (indicated with yellow triangles) on the dendrite can be visualized clearly after RTF clearing. Scale bar, 10 µm.

Supplementary Table 1. Clearing procedures of RTF

	Whole embryos or heads	Intact brains (E16-P12)	Sections (800-1500 µm)
RTF-R1 (30%TEA/40%F/30%W)	2-3hr	2-3hr	30-50min
RTF-R2 (60%TEA/25%F/15%W)	2-3hr	2-3hr	1-1.5hr
RTF-R3 (70%TEA/15%F/15%W)	5-14hr;E11-E15, respectively	O/N-1day;E16-P12, respectively	1-1.5hr

RTF clearing procedure includes graded concentration of solutions composed of triethanolamine and formamide. The incubation time in each solution varies with tissue type and thickness of samples. Time in final buffer can be determined by visual inspection for desired transparency. O/N, overnight. The brain slices of less than 800 µm can be incubated in only RTF-R2 just for 1-2hr. The size change of samples depends on incubation time, which can be adjusted for size maintainence according to practical needs.

Supplementary Table 2. Characteristics of RTF agents

Agents	Refractive Index	pH
RTF-R1	1.43	9.5
RTF-R2	1.45	10
RTF-R3	1.46	10

The refractive indices of RTF solutions increase with the concentration of triethanolamine. The agents are basic, with pH values of 9.5 or larger. The higher refractive index and alkalinity should be benefit for tissue transparency and fluorescence preservation.
